# The association of treatment with hydroxychloroquine and hospital mortality in COVID-19 patients

**DOI:** 10.1007/s11739-020-02505-x

**Published:** 2020-09-30

**Authors:** Luis Ayerbe, Carlos Risco-Risco, Salma Ayis

**Affiliations:** 1 Centre of Primary Care and Public Health Queen Mary University of London, 58 Turner Street, London, E1 2AB UK; 2 Carnarvon Medical Centre, Southend-on-Sea, UK; 3 Service of Internal Medicine. Hospital, Universitario HM Sanchinarro, Madrid, Spain; 4 School of Population Health and Environmental Sciences, King’s College London, London, UK; 5 National Institute for Health Research Biomedical Research Centre, Guy’s and St Thomas’ NHS Foundation Trust, London, UK

**Keywords:** Coronavirus infections, COVID-19, Hydroxychloroquine, Mortality

## Abstract

This study investigates the association between the treatment with hydroxychloroquine and mortality in patients admitted with COVID-19. Routinely recorded, clinical data, up to the 24th of April 2020, from the 2075 patients with COVID-19, admitted in 17 hospitals in Spain between the 1st of March and the 20th of April 2020 were used. The following variables were extracted for this study: age, gender, temperature, and saturation of oxygen on admission, treatment with hydroxychloroquine, azithromycin, heparin, steroids, tocilizumab, a combination of lopinavir with ritonavir, and oseltamivir, together with data on mortality. Multivariable logistic regression models were used to investigate the associations. At the time of collecting the data, 301 patients had died, 1449 had been discharged home from the hospitals, 240 were still admitted, and 85 had been transferred to hospitals not included in the study. Median follow-up time was 8 (IQR 5–12) days. Hydroxychloroquine had been used in 1857 patients. Hydroxychloroquine was associated with lower mortality when the model was adjusted for age and gender, with OR (95% CI): 0.44 (0.29–0.67). This association remained significant when saturation of oxygen < 90% and temperature > 37 °C were added to de model with OR 0.45 (0.30–0.68) *p* < 0.001, and also when all the other drugs, and time of admission, were included as covariates. The association between hydroxychloroquine and lower mortality observed in this study can be acknowledged by clinicians in hospitals and in the community. Randomized-controlled trials to assess the causal effects of hydroxychloroquine in different therapeutic regimes are required.

## Introduction

In December 2019, an outbreak of COVID-19, a novel disease caused by the virus SARS-CoV-2, was declared in China. In the first months of 2020, COVID-19 spread throughout the world [1, 2]. The number of new cases in some areas of the world is currently declining; however, it continues to rise worldwide with many countries having second outbreaks [3]. It has been estimated that 80% of affected patients have mild symptoms, and in the rest, hospital care can be necessary, and mortality is less than 2% [2, 4, 5]. The large number of simultaneous cases of COVID-19 has overloaded hospitals in many countries, making it very difficult to provide an adequate care. Many governments have used costly and disruptive policies to confine and distance the population to reduce the transmission of the disease and prevent a second wave [5–7]. These measures will need to be maintained in some manner until vaccines or effective treatments become available to avoid the risk of later epidemics [5, 8]. COVID-19 is negatively affecting all areas of healthcare, and is also having an adverse impact on the entire society and the international economy.

Using limited evidence and clinical experience, doctors have treated COVID-19 patients with different drugs to eliminate or reduce the presence of the virus, including hydroxychloroquine (HCQ) [9–12]. The use of this drug is based on its anti-inflammatory and antiviral effect [11, 12]. There are some in vitro data supporting the ability of HCQ to inhibit SARS-CoV-2 activity [13, 14].

HCQ may lead to the clinical improvement of the patients, it is safe, economical, and easy to use, and it can be given both to admitted and outpatients. If the treatment with HCQ is confirmed to be effective, it could be used in the early stages of the disease, and decrease the need for admissions, the mortality, the transmission of the infection, and its impact on other areas of health care. However, the use of HCQ is supported by limited evidence, and it may not lead to any clinical benefit. Studies on the clinical outcomes associated with HCQ are required. Research policies are currently shifting towards the investigation of more expensive treatments [15–18]. Studies on HCQ could inform evidence-based and affordable management for COVID-19, which may be particularly relevant when the world is facing an economic crisis, more so in areas where health care is based on limited resources.

This study investigates the association between treatment with HCQ and mortality, in hospitalized patients with COVID-19.

## Methods

The clinical records up to the 24th of April 2020, of all the patients with COVID-19 (*n* = 2075), admitted in all 17 Spanish hospitals, of the private healthcare provider HM Hospitales, were reviewed. These hospitals are based in the provinces of Barcelona, Coruña, León, Madrid, Pontevedra, and Toledo [19]. Patients had been diagnosed with polymerase chain reaction test of respiratory samples for SARS-CoV-2, between the 1st of March and the 20th of April 2020. Seven patients had been admitted before the 1st of March 2020. Data on age and gender, together with temperature and saturation of oxygen at the time of admission, were collected. Data on treatments at any time during admission with HCQ were collected. Once it was approved for clinical use in COVID-19 patients, HCQ was started immediately after admission, and it was stopped if any abnormalities were identified in the ECGs that were done on a daily basis to patients who were taking it. HCQ was dosed as 400 mg twice daily the first day, followed by 200 mg twice daily for 4–6 days. Data on treatments with azithromycin, steroids, heparin, tocilizumab, a combination of lopinavir with ritonavir, and oseltamivir, were collected, as well. The limited evidence on which these treatments were based, and the rapidly evolving clinical protocols, resulted in these drugs being given in many different specific preparations, doses, and frequency. No information on specific preparations of these drugs, dosage, duration of treatment, or route of administration, were collected. Finally, data on death were recorded.

The age of patients treated and not treated with HCQ was compared with t tests. The proportion of men and women for those treated and not treated with HCQ was compared with Chi-squared tests. The association between treatment with HCQ and mortality was examined with four different logistic regression models: model one was adjusted for age and gender; model two included age and gender, together with temperature > 37 °C, and saturation of oxygen < 90% on admission, which were both associated with mortality in an exploratory analysis; model three had all the variables previously mentioned together with treatment with azithromycin, steroids, heparin, tocilizumab, a combination of lopinavir with ritonavir, and oseltamivir; finally, to account for the change in clinical management during the study period, model four was adjusted for all the previously mentioned demographic, clinical severity measures, and drugs, together with a categorical variable for date of admission (before the 10th of March, 11–20th of March, 20–31st March, 1st–10th of April, and 11–20th of April). All covariates included in the four models were considered potential confounders. No further variables were included to avoid the complex interpretation of results and collinearity. The software Stata 14.0 was used for the analysis [20]. Missing data were treated as a separate category for temperature and oxygen saturation. Sensitivity analyses were made to compare estimates based on using missing data as categories with, (1) estimates based on complete data dropping variables with missing observations, and (2) complete case analysis; dropping cases with missing data.

## Results

Among the 2075 patients whose records were reviewed, 1256 were men, 819 were women, and the mean age was 67.57. At the time of collecting the data, 301 had died, 1449 patients had been discharged home from hospitals, 240 were still admitted, and 85 had been transferred to hospitals not included in the study. Median (IQR) follow-up time was 8 (5–12) days. Data on treatment with HCQ were available for 2019 patients. There was a younger age (*p* < 0.001), and a higher proportion of men (*p* = 0.017) for those who received HCQ. Among the 1857 patients who had been treated with HCQ, 237 had died. (Table 1).

**Table 1:** Description of cohort

	*N*	Age mean (SD)	Female *n* (%)	Death *n* (%)
Total cohort	2075	67.57 (15.52)	819 (39.47)	301 (14.51)
Oxygen saturation < 90%				
Yes	70	73.18 (13.97)	20 (28.57)	28 (40.00)
No	221	67.00 (16.14)	95 (42.99)	24 (10.86)
Temperature > 37 °C				
Yes	159	61.20 (17.27)	47 (29.56)	24 (15.09)
No	1422	68.53 (15.65)	577 (40.58)	197 (13.85)
Admission before 10th March	40	66.90 (16.57)	11 (27.50)	9(22.50)
Admission 10–19th March	412	64.73 (15.52)	156 (37.86)	82(19.90)
Admission 20–31st March	946	65.88 (14.63)	338 (35.73)	126(13.32)
Admission 1st–10th April	449	70.56 (16.78)	206 (45.88)	62(13.81)
Admission 11–20th April	228	73.93 (17.37)	108 (47.37)	22(9.65)
HCQ				
Yes	1857	67.11 (15.51)	705 (37.96)	237 (12.76)
No	162	73.47 (16.22)	77 (47.53)	49 (30.25)
Azithromycin				
Yes	1223	68.33 (15.03)	456 (37.29)	146 (11.94)
No	796	66.54 (16.52)	326 (40.95)	140 (17.59)
Steroids				
Yes	960	69.88 (14.01)	330 (34.38)	200 (20.83)
No	1059	65.58 (16.76)	452 (42.68)	86 (8.12)
Heparin				
Yes	1734	68.59 (15.09)	686 (39.56)	242 (13.96)
No	285	61.76 (17.67)	96 (33.68)	44 (15.44)
Tocilizumab				
Yes	421	66.19 (13.11)	117 (27.79)	89 (21.14)
No	1598	68.00 (16.24)	665 (41.61)	197 (12.33)
Lopinavir + ritonavir				
Yes	1230	63.94 (14.28)	421 (34.23)	160 (13.01)
No	789	73.37 (15.99)	361 (45.75)	126 (15.97)
Oseltamivir				
Yes	132	67.78 (13.79)	51 (38.64)	26 (19.70)
No	1887	67.61 (15.78)	731 (38.74)	260 (13.78)

HCQ was associated with lower mortality when the model was adjusted for age and gender with OR: 0.44 (0.29–0.67), *p* value < 0.001. When the model was adjusted for age, gender, together with temperature > 37 °C and saturation of oxygen < 90% on admission, and also when the model included all the previous variables plus treatment with all drugs, the association between use of HCQ and lower mortality remained significant. The analysis of interaction suggested that HCQ was associated with lower mortality, and there was no difference for those taking (*n* = 1187), or not (*n* = 670), azithromycin, as well. In the final model where all the previous demographic, clinical variables, and drugs were introduced, together with time of admission, the association between treatment with HCQ and lower mortality was also significant. (Table 2).

**Table 2:** Association between HCQ and mortality

	Mortality odds ratio (95% CI)	*p* value
Model 1	0.44 (0.29–0.67)	< 0.001
Model 2	0.45 (0.30–0.68)	< 0.001
Model 3	0.39 (0.24–0.64)	< 0.001
Model 3 with a statistical interaction term for HCQ and azithromycin		
1 Main effect HCQ	0.56(0.34–0.92)	0.022
2 Main effect azithromycin	0.53(0.19–1.50)	0.233
3 Interaction (1 and 2)	1.11(0.38–3.29)	0.846
Model 4	0.39 (0.24–0.64)	< 0.001

Model 1 Adjusted for age and gender

Model 2 Adjusted for age, gender, temperature > 37 °C, and saturation of oxygen < 90%

Model 3 Adjusted for age, gender, temperature > 37 °C, and saturation of oxygen < 90% treatment with azithromycin, steroids, heparin, tocilizumab, a combination of lopinavir with ritonavir, and oseltamivir

Model 4 Adjusted for age, gender, temperature > 37 °C, and saturation of oxygen < 90% treatment with azithromycin, steroids, heparin, tocilizumab, a combination of lopinavir with ritonavir, and oseltamivir, and date of admission

## Discussion

Treatment with HCQ was associated with lower mortality in patients admitted with COVID-19.

This study has strengths and limitations. Patients were not randomized and the differences in mortality may be explained by factors other than the use of HCQ. In an effort to control for the severity of disease, available markers were considered, and adjusted for, in all the models. The observation of a large number of unselected patients admitted in 17 hospitals, and the analyses run with some variations to test the consistency of the results are also strengths of this study. However, residual confounding is always present in all observational research. The association, that was consistent across a set of models that adjusted for different potential confounders, provides support to an independent positive effect of HCQ. For all models, the sensitivity analyses described in methods were used to assess the impact of missing data on the two markers of disease severity, and results were consistent with those presented.

HCQ has been used in all phases of COVID-19 since two studies, with no control arm and small sample size, published in March 2020, reported it to be associated with a reduction of the viral carriage and clinical improvement [9, 10, 21]. This has allowed for its effects to be observed since then in a large number of different patients in many countries. Our results, showing that HCQ is associated with positive outcomes, are consistent with the ones first reported in March 2020. A number of observational studies later conducted in China, France, Spain (in a hospital not included in our project), and the USA, have also reported the association between HCQ and lower mortality [22–27]. Three of these studies included over 2000 participants [23, 24, 26]. In one of these studies, only patients having mechanical ventilation were included [22]. In another one, HCQ was also associated with lower probability of admission to intensive care, shorter hospital admissions, and shorter duration of viral shedding [23]. In two of these studies, favourable results of HCQ combined with azithromycin were reported [23, 24], and in another one, the lower mortality was observed for those on HCQ in combination with azithromycin and zinc [27]. The later study was based on primary care, and reported an association between treatment and lower rates of hospital admissions, as well. Another observational study conducted in primary care, were mortality was not the outcome, showed significantly shorter time to clinical recovery for those treated with HCQ or HCQ plus azithromycin [28]. In our study, the addition of azithromycin to HCQ does not seem to add any clear benefit. A large multicentre observational study reported no association between HCQ, or HCQ with azithromycin, and lower mortality but significantly higher rates of discharge home were observed for patients treated by HCQ [29].

In a recent meta-analysis, studies were classified as big data, when electronic medical records had been used, or clinical studies, when details of treatments were reported and the study had been conducted by the same physicians who cared for the patients. It reported, among clinical studies, an association of chloroquine derivates with clinical improvement, lower mortality, and viral carriage [30]. Finally, a small RCT reported the association of HCQ with shorter time both to clinical recovery and to reach viral RNA negativity [31].

The absence of any positive effects of the treatment with HCQ has also been reported. A number of observational studies have reported that HCQ, either alone or in combination with azithromycin, was not associated with lower mortality [32–35]. Three of these studies included over 1000 participants [32, 34, 35]. One of these studies included patients who needed oxygen [33] and another one used mortality or need for intubation as an outcome [34] Two RCTs have also reported no association between HCQ and survival [36, 37]. One of them also reported no benefit in need for admissions or clinical improvement [37]. The lack of clinical improvement was reported in another trial [38]. Finally, two more RCTs have reported no association between HCQ and virological clearance [39] or prevention of disease in individuals exposed to it [40]. A number of factors could explain the difference between our results and the ones observed in these studies [32–40] including the following: the clinical–epidemiological design of our work; [30] the involvement of all patients admitted with COVID-19, regardless their past medical history, the time between onset of symptoms and the start of treatment, the duration of admission, and the need for oxygen; the different statistical approach; and the observation in our work of patients from private hospitals, who are likely to have a high socioeconomic status [41]. The safety of the HCQ has been questioned, as it could negatively impact the immune response to the virus, or cause abnormalities in the ECG [33, 42, 43]. However, none of the studies that we have reviewed, reporting no benefit on HCQ, show an increased mortality associated with it [32–40].

Further RCTs, observational studies, and summaries of both types of evidence to assess the associations between HCQ and survival are necessary. Future studies could also address at what dosage, and in what phase of the disease, does HCQ lead to the best possible outcome, for patients with different past medical histories [44]. The interventional evidence on the management of COVID-19 is still limited. Therefore, clinicians could acknowledge the results presented in this study. The positive effect of HCQ seems consistent and its use could be considered in clinical settings. HCQ is easy to administer, and its use in ambulatory patients, to reduce symptoms, prevent admissions, decrease mortality, and the transmission of the disease, could also be considered by clinicians and future researchers.

## Data Availability

Data requests should be addressed to HM Hospitales on: data_science@hmhospitales.com or coviddatasavelives@hmhospitales.com. The corresponding author would also make the datasets used in this study available for verification once permission from HM Hospitales is granted. The Stata do files used in this study are available from corresponding author.
